# Supplementary material for: Dysbiosis of Oral Microbiota and Metabolite Profiles Associated with Type 2 Diabetes Mellitus
Source: Microbiol Spectr. 2023 Jan 10;11(1):e03796-22. doi: 10.1128/spectrum.03796-22 (PMC9927158; doi:10.1128/spectrum.03796-22)
Supplement: Supplemental file 1 — Fig. S1 to S4 and legends for Tables S1 to S5. Download spectrum.03796-22-s0001.pdf, PDF file, 0.4 MB [file spectrum.03796-22-s0001.pdf]

Supplemental materials

Supplementary Figures

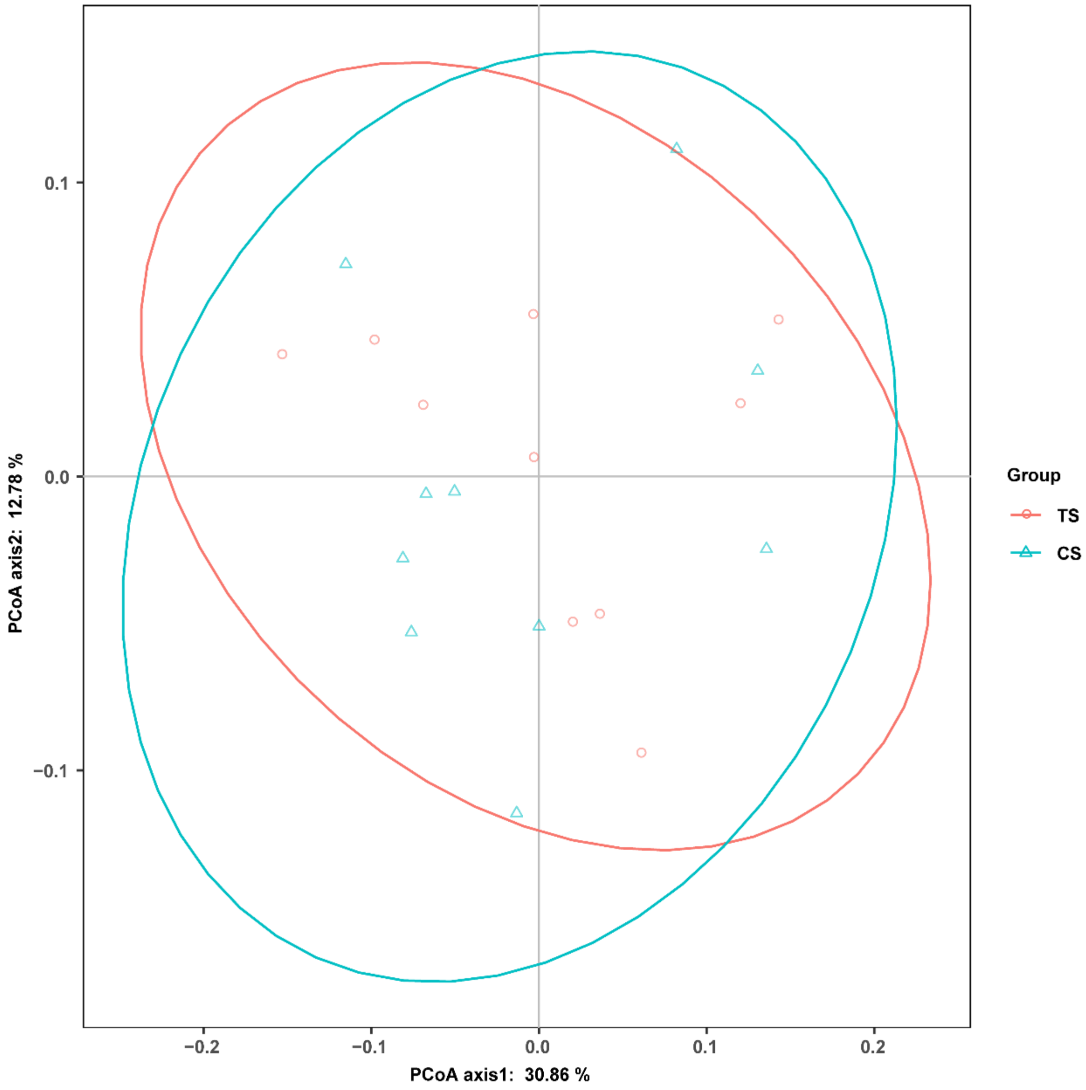

Supplementary Figure 1 PCoA analysis of salivary microbial community between T2DM group and normal control group. Data points in different shapes and colors represent for samples in variant conditions. Scales of X-axis and Y-axis are the projection of samples' coordinates in 2-dimension. PCoA axis1 and PCoA axis2 stand for the possible factors that drive the changes of microbiota structure in groups, which needs to be interpreted in combination with groups features.

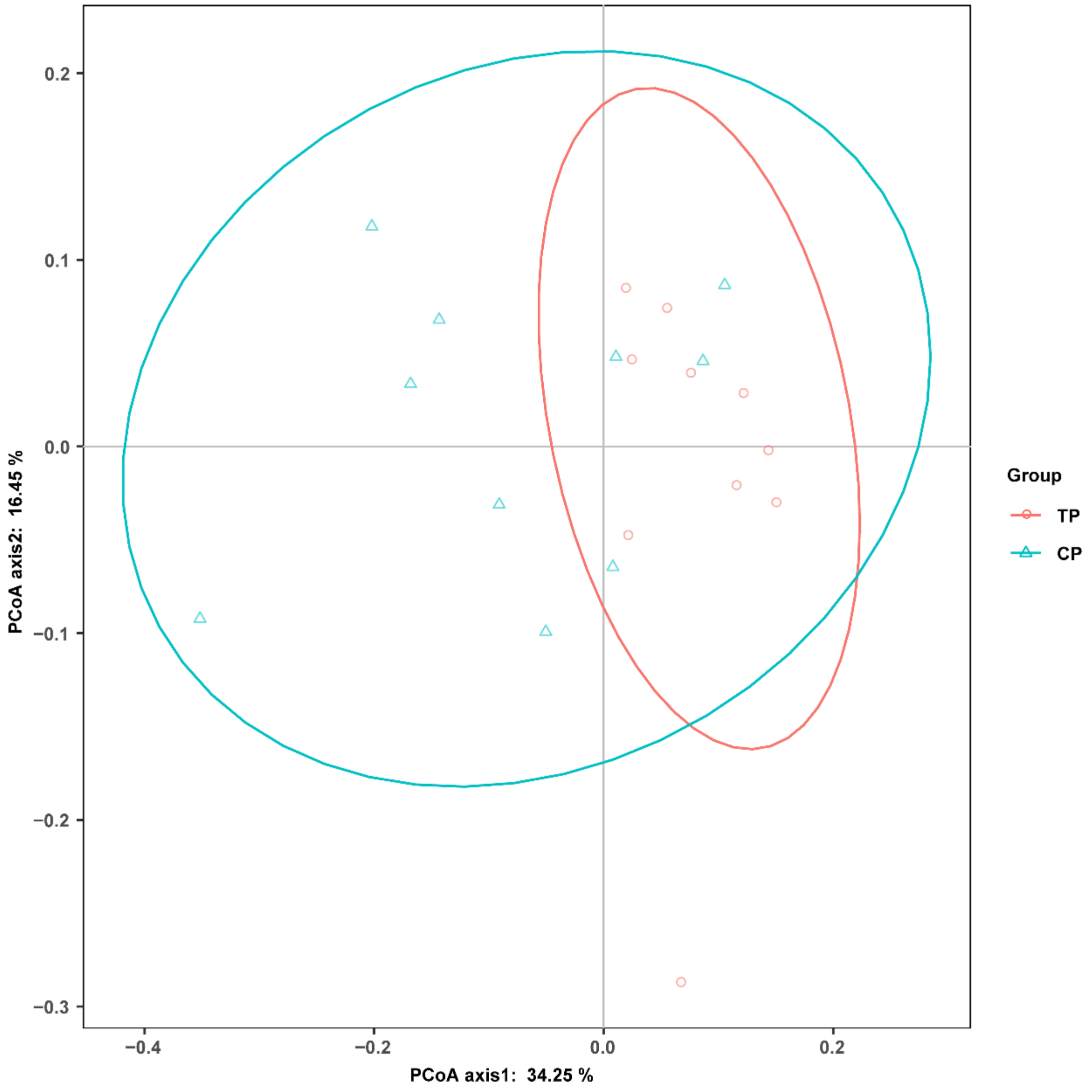

Supplementary Figure 2 PCoA analysis of supragingival plaque microbial community between T2DM group and normal control group. Data points in different shapes and colors represent for samples in variant conditions. Scales of X-axis and Y-axis are the projection of samples' coordinates in 2-dimension. PCoA axis1 and PCoA axis2 stand for the possible factors that drive the changes of microbiota structure in groups, which needs to be interpreted in combination with groups features.

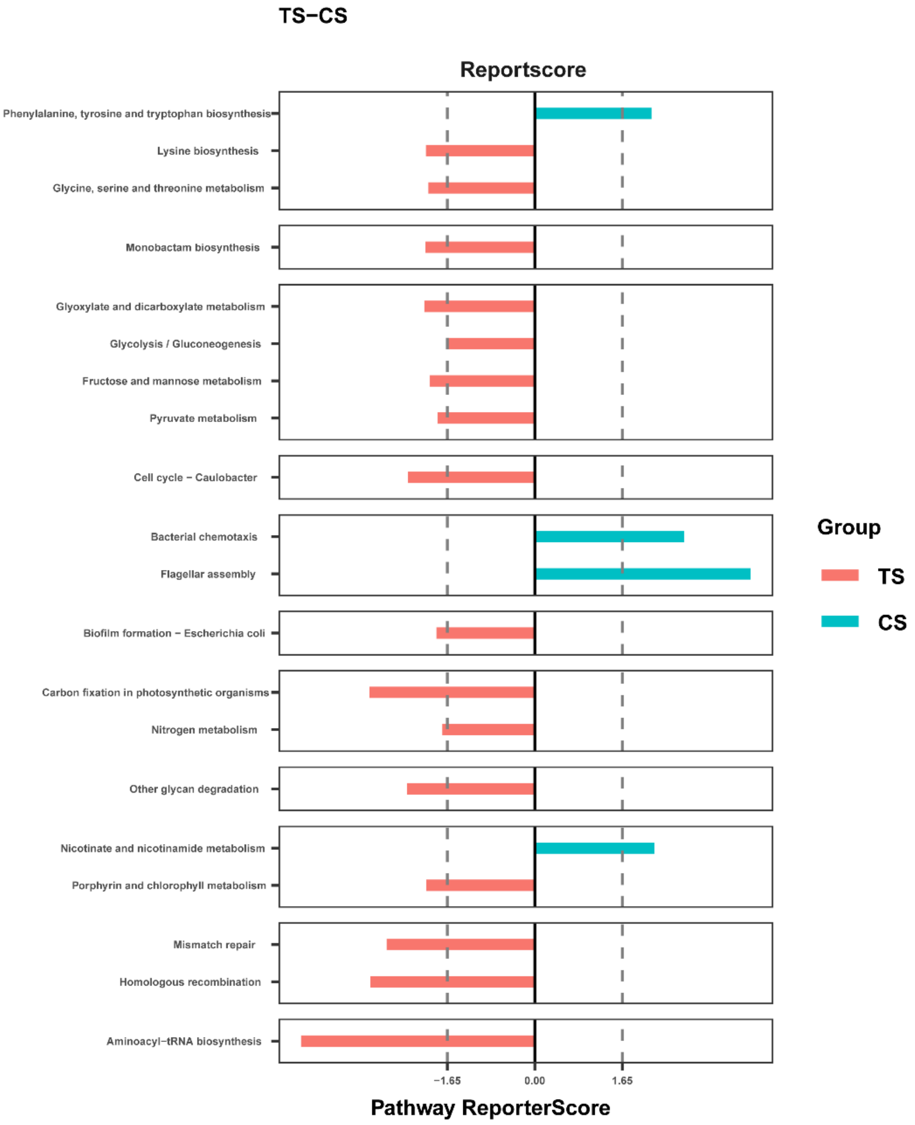

Supplementary Figure 3 KEGG metabolic pathway enrichment map of salivary microorganisms between T2DM group and normal control group. X-axis shows reporterscore values, small blocks on Y-axis show pathways. Differences are significant when bars exceed dashed lines.

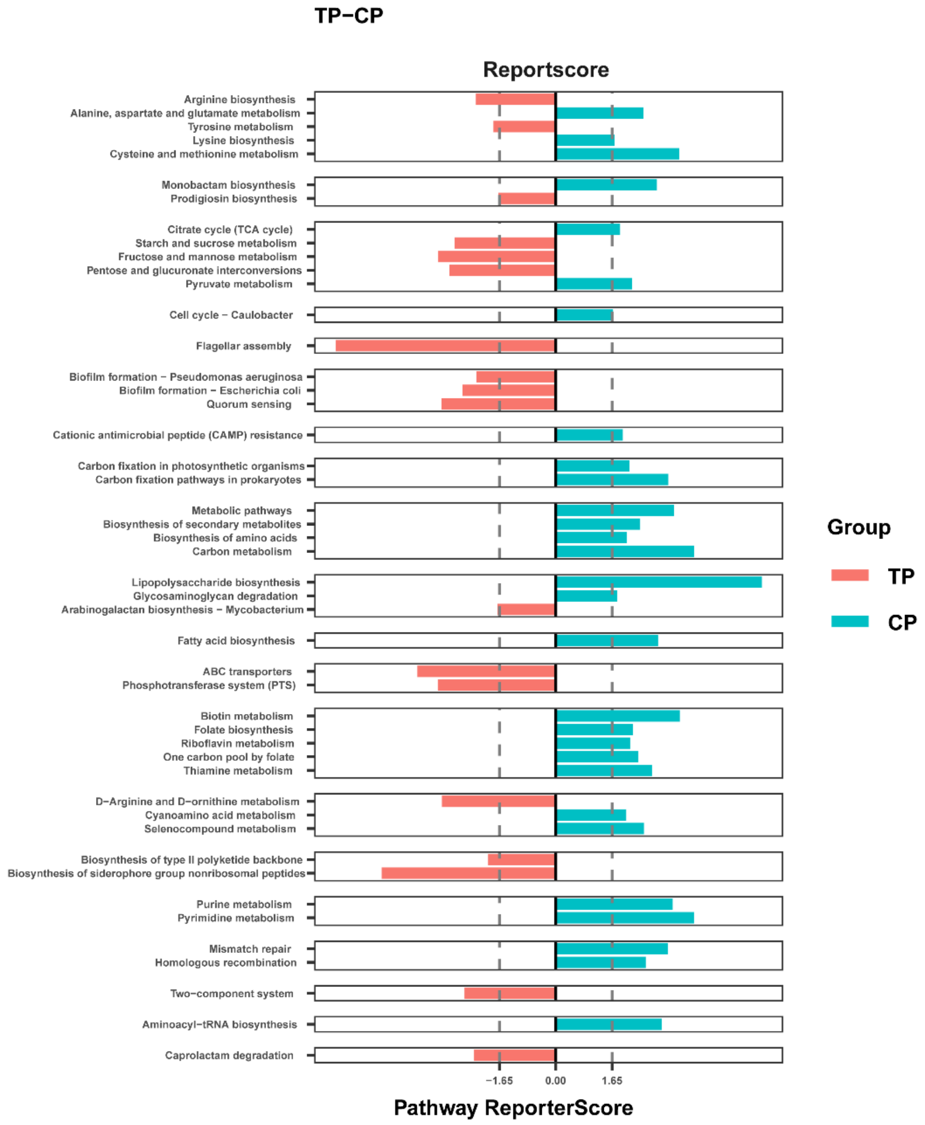

Supplementary Figure 4 KEGG metabolic pathway enrichment map of supragingival plaque microorganisms between T2DM group and normal control group. X-axis shows reporterscore values, small blocks on Y-axis show pathways. Differences are significant when bars exceed dashed lines.

Supplementary Table S1: Salivary differential metabolites identified in POS and NEG models between T2DM group and normal control group.

Supplementary Table S2: Supragingival plaque differential metabolites identified in POS and NEG models between T2DM group and normal control group.

Supplementary Table S3: Salivary differential metabolic pathways identified in POS and NEG models between T2DM group and normal control group.

Supplementary Table S4: Supragingival plaque differential metabolic pathways identified in POS and NEG models between T2DM group and normal control group.

Supplementary Table S5: Spearman's correlation analysis between metabolic patterns and species.
